# THE AGING NETWORK AS A CATALYST FOR COMMUNITY GERONTOLOGY AND APPLIED RESEARCH

**DOI:** 10.1093/geroni/igad104.0839

**Published:** 2023-12-21

**Authors:** Traci Wilson, Emily Greenfield

## Abstract

The Older Americans Act (OAA) established Area Agencies on Aging (AAAs) fifty years ago as local community-based organizations responsible for planning, coordinating, funding, and delivering nutrition and other supportive services which allow older adults to remain living in their homes and communities. Local control and decision-making, mandated by the OAA, has allowed AAAs to adapt to the evolving needs and demands of their communities The centrality of their role was never more apparent than during the COVID-19 pandemic. As national and societal circumstances have resulted in increased social isolation, housing insecurity, and public health concerns for older adults, AAAs have adapted and expanded beyond traditional OAA services to address the social and health needs of an increasingly diverse older adult population. The Community Gerontology framework provides a useful structure to understand the evolution and impact of AAAs and the broader Aging Network on the health of older adults. Using data from the 2022 National Survey of AAAs and a new qualitative study on Community Care Hubs, academic and Aging Network-based researchers will illustrate the many partnerships and pathways of mutual influence between AAAs and the micro and macro levels to describe how Aging Network services are expanding provision of services for health care partners, public health initiatives, and new housing and homelessness services and partnerships to address emerging needs. The symposium will conclude with an illustration of how applied researchers and Aging Network organizations partnered on research-informed practice to innovate and strengthen services for at-risk older adults.

